# Development of a High Strength Geopolymer Incorporating Quarry Waste Diabase Mud (DM) and Ground Granulated Blast-Furnace Slag (GGBS)

**DOI:** 10.3390/ma15175946

**Published:** 2022-08-28

**Authors:** Thomaida Polydorou, Maria Spanou, Pericles Savva, Konstantinos Sakkas, Konstantina Oikonomopoulou, Michael F. Petrou, Demetris Nicolaides

**Affiliations:** 1 Department of Civil and Environmental Engineering, University of Cyprus, 75 Kallipoleos Av., P.O. Box 20537, Nicosia 1678, Cyprus; 2 Frederick Research Center, P.O. Box 24729, Nicosia 1303, Cyprus; 3 Latomia Pharmakas, 23 Themistokli Dervi Av., S.TA.D.Y.L. Building, P.O. Box 23504, Nicosia 1066, Cyprus; 4 RECS Engineering, Griva Digeni Av., Lakatamia, Nicosia 2450, Cyprus

**Keywords:** waste diabase mud, GGBS, cementless binders, geopolymer binders, alkali activated materials, materials valorization, sustainability, circular economy

## Abstract

This study presents the development and experimental assessment of novel, high strength, cementless binders that incorporate alkali-activated local waste. A silica-rich diabase mud (DM), currently considered as waste, was previously investigated for geopolymerization, signifying that the DM lacked the necessary reactivity to provide a stable geopolymer binder alone. Moreover, even after incorporation of small amounts of cement and metakaolin, the DM mixtures still did not yield adequate mechanical properties. In this study, the local DM was instead combined with another industrial byproduct known as Ground Granulated Blast-furnace Slag (GGBS) in varying mixtures. The mixture design trials enabled the development of three high strength cementless geopolymer mixtures with 28-day compressive strengths ranging between 60 and 100 MPa, comparable to conventional concrete compressive strengths. The results indicate that the innovative geopolymer material is very promising for the manufacturing of pavement tiles and other precast construction products. Most importantly, this study presents the first successful development of a construction material of adequate compressive strength that can absorb large quantities of the abundant quarry waste, following a course of 10 years of unsuccessful attempts to valorize the local DM. Although difficulties were encountered due to a high reactivity rate, especially for the mix that included the highest GGBS content, prototype pavement tiles were manufactured and assessed experimentally. The results reveal a promising potential of valorizing the local DM in the development of precast geopolymer products, despite the effects of shrinkage cracking on the experimental evaluation of the material mechanical properties.

## 1. Introduction

Environmental concerns regarding the cement and concrete contribution to CO_2_ emissions in the atmosphere have captured the attention of researchers, who have recently turned the focus onto reducing cement content in concrete without compromising strength, if possible. In conventional concrete mixtures, cement is partially replaced by industrial byproducts such as fly ash (FA), silica fume (SF), metakaolin (MK) and ground granulated blast-furnace slag (GGBS). Furthermore, aiming to reduce the use of cement use in construction, the development of eco-friendly, cementless alternatives to concrete has been rising, leading to significant advancements in geopolymer research [1].

Geopolymer research and development provides the revolutionary opportunity to reuse waste into novel building material mixtures, while concurrently contributing to decreasing the detrimental environmental impact imposed by the construction industry mainly due to the extremely high temperatures required for cement manufacturing. Cement accounts for roughly 8% of anthropogenic CO_2_ emissions [2,3] and is the principal constituent of concrete, which is currently the most widely used construction material [4,5] as well as the highest consumed substance in the world after water [4,6].

Following the circular economy principles [7], which entail extending the life cycle of products and reducing waste to a minimum, locally available waste is exploited for inclusion in novel construction material mixtures [6]. Moreover, byproducts of the quarry industries, which constitute a major environmental and health threat globally due to exponentially increasing accumulation, could be effectively reused in construction materials [8]. The new circular economy action plan (CEAP) adopted by the European Commission in March 2020 [9] includes targets for landfill reuse to create further value, in line with the European Green Deal [10] goals for sustainable economic growth.

Geopolymers are alkali-activated aluminosilicates, integrating silica-rich industrial byproducts and wastes that are activated using alkaline solutions such as NaOH, potassium hydroxide and calcium hydroxide among others [1,11]. In addition to industrial byproducts such as fly ash (FA), metakaolin (MK), silica fume (SF) and ground granulated blast-furnace slag (GGBS), which have been most commonly used as partial cement replacement in concrete technology, geopolymer mixtures can incorporate additional types of wastes such as waste clay brick powder [1,12], glass polishing waste [1,13], palm oil fuel ash (POFA) [14] and even rice husk [1], a silica-rich waste material, abundantly available in all rice producing countries [15,16]. Previous research studies have denoted the potential of geopolymer mixtures to reach higher compressive strengths than conventional concrete [17] and possess fracture energy comparable to cement concrete [14,18] as well as superior durability [19]. Therefore, a silica-rich waste mud, produced in abundance by the quarry industry on the island of Cyprus, was investigated for geopolymerization.

During the last 10 years, various studies [19,20] in Cyprus have attempted to develop a cost-effective way to valorize the particular hazardous DM waste. Even though several cases have been investigated, none of them were successful. In the meantime, accumulation of DM has increased, and certain quarries are presented with the challenge of DM storage, with their production exceeding 25,000 tons yearly. Bearing in mind the availability and environmental issues pertaining to DM accumulation, research continues to investigate effective reuse of the waste DM in construction materials [6].

Previous research revealed the significant SiO_2_ content (nearly 41%) of the local quarry waste “Diabase Mud” (DM), deeming it worthy of geopolymerization, but dissolution test results indicated limited reactivity potential denoting that the DM alone is not capable of producing chemically stable alkali activated binders. Alkali-activation was therefore implemented in combination with CEM I cement, Gypsum and Metakaolin (MK), but none of the mixtures were able to reach compressive strengths higher than 10 MPa [19]. To further explore valorization of the local waste DM in geopolymer development, this research study focused on alkali-activating the DM along with the industrial byproduct GGBS.

A byproduct of the iron manufacturing industry blast furnaces, GGBS is rich in calcium oxide and silica, and of similar density to ordinary cement. It is commonly used as cement replacement at ready mix concrete plants for sustainability. In geopolymers the use of GGBS allows for ambient curing conditions [21,22], whereas under other circumstances high temperature curing is necessary for alkali activation. In addition, GGBS has indicated an ability to significantly accelerate reactivity of activator solutions, therefore geopolymer mortars that incorporate high GGBS percentages are expected to attain reduced workability [21]. Although researchers were able to alleviate workability issues by incorporating metakaolin (MK) instead of GGBS [14], this study aims to provide a cementless binder for large-scale manufacturing of precast construction products, thus availability and cost of materials were considered and therefore the use of GGBS was largely favorable to Metakaolin (MK) due to cost-effectiveness.

Mechanical properties of three mixtures with varying DM/GGBS content combinations were examined. In addition, full scale pavement tiles were manufactured and tested according to standard procedures, as a first attempt in the development of a novel cementless binder, capable of being produced on a larger scale and intended for the industrial manufacturing of precast products (e.g., pavement tiles) for the local and international market that will not only be more sustainable and cost effective, but will possess equivalent or even superior properties compared to conventional market products.

## 2. Materials and Methods

This study investigates the development of high compressive strength cementless binder mixtures incorporating local waste DM and GGBS. The DM is a byproduct of local diabase quarries, specifically the sludge derived from the remainders of aggregate production processes of crushing and washing. The DM consists mostly of silicon and alumina oxides and its original sludge form contains about 25–30% water. At nearly 41% quartz or silicon dioxide, the crystalline composition of the waste DM revealed significant potential for geopolymerization. The detailed oxide composition of the DM, as obtained through Energy Dispersive X-Ray Fluorescence (ED-XRF) analysis [19], is presented in Table 1.

The DM average density was measured after oven drying the as-received sludge at 105 °C until mass stability was reached and subsequently grinding to particle sizes smaller than 63 microns. The results indicated a 2362 kg/m^3^ average density, with a standard deviation of 25.2 kg/m^3^. The experimentally obtained material density was slightly lower than the reported average density of crushed diabase aggregates produced by the specific quarry that supplied the DM [19].

To determine the reactivity potential of the DM, dissolution testing was conducted using NaOH and KOH at different molarities, up to 16M [19]. The low amount of either Al or Si leaching indicated that the DM alone may not be dissolved sufficiently in any one of the two alkaline activators used, even at high molarities. To enable successful activation of a single raw material, leading to stable geopolymer development, a minimum 10% dissolution is required while as reported by various studies, higher raw material dissolution percentages lead to superior geopolymer mechanical properties. The DM investigated in this study had a maximum dissolution of 2.54% at 12M NaOH and is therefore considered unreactive and unable to be alkali-activated alone [19].

Since the DM reactivity is not sufficient for geopolymerization unless combined with other raw materials, and previous attempts to activate it with CEM I, gypsum and MK did not yield binders of adequate compressive strength, incorporation of GGBS is investigated in this study. GGBS mainly consisting of CaO (43.8%), SiO2 (37.7%), Al_2_O_3_ (10.2%) and MgO (6.4%) was used to assist in DM activation [23]. The GGBS technical data [23] conveyed by the GGBS provider are summarized in Table 2. In addition to the properties reported in Table 2, indicative compressive strengths and setting times were given by the GGBS provider, namely compressive strength and setting time results for a 50% GGBS-50% Cement paste and a control 100% Cement paste, for comparison purposes. Therefore, according to the values provided [23], replacing 50% of the Cement included in a 100% Cement control mixture by the GGBS utilized in this study led to slight reduction in both the 7- and 28-day compressive strengths but did not affect the 90-day compressive strength, while it also delayed setting by 35 minutes, or increased setting time by 20% [23].

In this study, 3 mixtures were developed and assessed experimentally. The 3 mix-tures incorporated DM and GGBS at varying contents, mixed with equal amounts of liquid Na_3_SiO_2_ and 8M NaOH. To reduce the cost of processing, the DM was used in the sludge form received, but to keep the moisture content consistent throughout the study the DM was allowed to release part of its moisture content (while being monitored) and was used in mixture development while at a moisture content of 22% in all cases. The mixtures are labeled according to their DM and GGBS content; 70%DM-30%GGBS, 50%DM-50%GGBS, and 30%DM-70%GGBS. The mix constituents per 1 m^3^ are shown in Table 3. It should be noted that even though a consistent S/L ratio of 5 was planned and used during the preliminary study, the S/L ratio of the 30%DM-70%GGBS mix had to be modified to 3.75 to enable casting of specimens within the workable time window. Higher GGBS content led to higher temperatures and quicker setting. This issue is further discussed in Section 3.1.

A standard mortar mixer was used for the mixture preparation, following standard mortar preparation procedures, but to accommodate for the fact that GGBS is known to react quickly with alkaline solutions leading to accelerated mix setting, the liquids Na_3_SiO_2_ and 8M NaOH were initially mixed together before the DM and finally the GGBS were added to the mixer. To examine the hardened mechanical properties of the 3 mixtures, 100 × 100 × 100 mm cubic, 40 × 40 × 160 mm prismatic and 200 × 100 mm cylindric specimens were cast in metal forms, while 400 × 400 × 40 mm pavement tiles were cast in wooden forms. The setting time, 7- and 28-day compressive strengths, modulus of elasticity, splitting tensile strength and flexural strength of flags as well as sorptivity were determined for the 3 DM–GGBS mixtures by experimentally evaluating that the 3 specimens were per mixture for all experimental testing procedures.

The setting time for the 3 geopolymer mixtures developed in this study was assessed following the guidelines for testing cement for determination of setting times [24]. The 3 geopolymer mixtures developed were also tested for the Rate of Absorption of Water (Sorptivity) following standard guidelines by Hydraulic Cement Concretes [25], with the only deviation from the standard being the fact that 100% concentrate Isopropyl alcohol (C_3_H_8_O) [26] was used instead of water.

The compressive strength of standard 100 × 100 × 100 mm cubes was determined at 7-days and 28-days maturity. Cubic specimens were cast following preparation guidelines for hardened concrete specimens intended for strength testing [27], but instead of following standard curing procedures for concrete the geopolymer cubes were cured in a controlled indoor environment at 30 °C while sealed in plastic bags to avoid sudden loss of moisture. The particular curing regime was selected based on preliminary studies on a variety of alternative curing options. Subsequently, the geopolymer cubes were tested for compressive strength following the hardened concrete testing guideline [28].

Similarly, splitting tensile strength of the 3 mixtures developed was evaluated following the Standard Test Method for Splitting Tensile Strength of Cylindrical Concrete Specimens [29]. In addition, the flexural strength of prismatic specimens was experimentally evaluated following the standard procedure for concrete [30], while likewise the modulus of elasticity values for each mixture were determined following standard guidelines for concrete specimens [31]. Finally, the flexural strength of paving flag specimens was examined following the standard guidelines for equivalent concrete products [32]. The results are presented and discussed in Section 3. Testing geopolymer mixtures using the conventional cementitious mixture standards is common practice [33], while Rilem Technical Committee 224-AAM is working on providing performance-based specifications and recommendations for the development of standards that will specifically apply to alkali-activated materials [34].

## 3. Results

### 3.1. Fresh Geopolymer Mixture Properties

**Figure 1:**
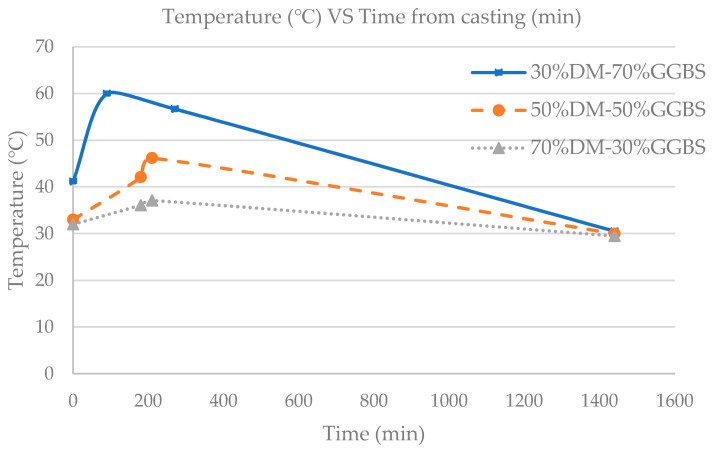
Internal temperature (°C) vs. Time from casting (min).

The mix temperature was monitored during preparation of the three DM/GGBS mixture combinations. As expected, the higher the GGBS content, the greater the rise in temperature. The temperature began to rise shortly after the GGBS was added to the mixer and is attributed to the chemical reaction between the GGBS and alkali-activator used due to the high content of reactive oxides being present in the GGBS, particularly CaO. As expected, the 30%DM-70%GGBS mix experienced an immediate rise in temperature due to higher GGBS content [21] prior to being placed into cube molds. Thermocouples were inserted into cubes as soon as the specimens were cast. The internal temperature over time was monitored for the three geopolymer mixtures for 24 h during the preliminary study, where all three mixtures had a constant S/L ratio of 5. The internal temperature values vs. time after casting are shown in Figure 1.

**Figure 2:**
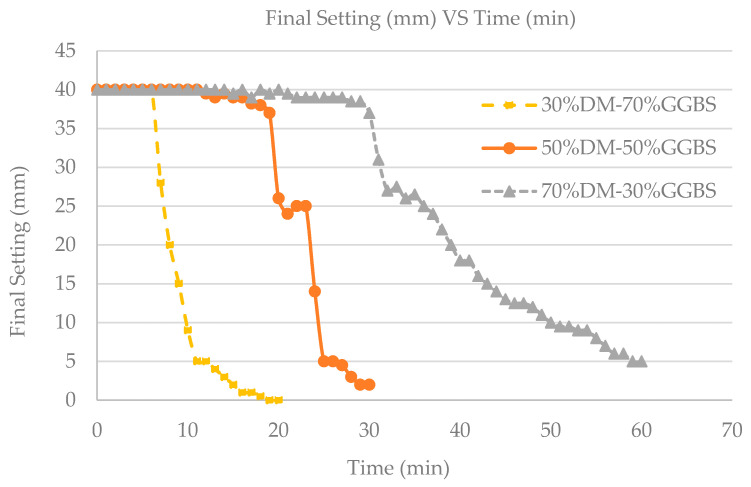
Setting (mm) vs. time (min) plots for the 3 DM–GGBS mixtures developed.

In addition to temperature rise, the rate of reactivity can affect mixture setting times. As shown in Figure 2, the geopolymer final setting time is reduced with increasing GGBS content. The early age microstructure and setting time of alkali activated binders incorporating GGBS is affected to a great extent by the chemical composition and fineness of the GGBS [35], which in our case is rich in CaO (43.8%), SiO_2_ (37.7%), Al_2_O_3_ (10.2%) and MgO (6.4%) and has a density of 2.90 ± 0.03 g/cm^3^ [23]. Increasing rate of reactivity and temperature rise, ultimately leading to a reduction in setting time observed within this study, were expected with increasing GGBS content. The final setting vs. time was monitored during the preliminary study where all mixtures had a consistent S/L ratio of 5.

As noted by previous research, geopolymer mixtures incorporating GGBS suffer from poor workability and could thus be enhanced by super plasticizing agents or substitution of GGBS by other industrial byproducts such as FA, SF and MK. Considering the objective of this research, which was to valorize the local DM by developing an affordable mixture intended for large scale industrial production of precast products, the use of expensive chemicals to improve mixture workability was avoided. The use of MK instead of GGBS was not considered either, since the DM was not found to produce compressive strengths over 10 MPa when activated during a previous study with MK even with the presence of CEM I [19]. In addition, MK is not readily available in Cyprus and would substantially increase the mixture cost if it were to be incorporated in the mixtures.

**Figure 3:**
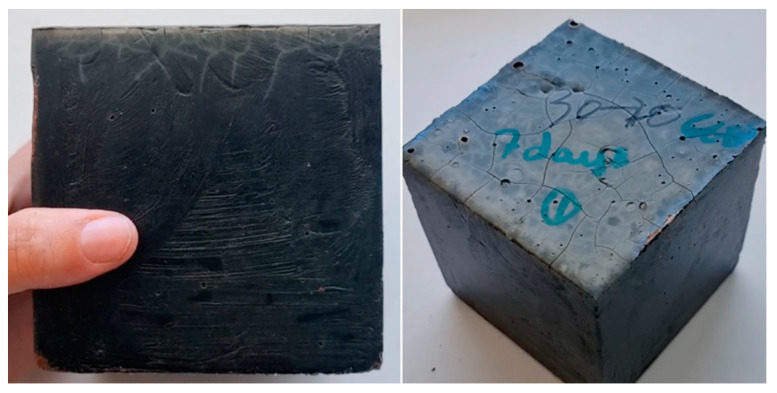
Preliminary study 30% DM-70% GGBS, 50 × 50 × 50 mm^3^ Cubic Specimen Images.

When small-scale mixtures were prepared for 50 × 50 × 50 mm cubic specimen preparation during the preliminary mix development trials, the setting time issue was not as critical since smaller specimens require less time and effort to be cast, therefore casting was feasible during the available time frame before the geopolymer mixtures hardened. It should also be mentioned that the heat generated by the reaction was perceived to increase with increasing mix volume, even though no data are available for comparison. The higher temperature rise and shorter workable time frame associated with the higher GGBS content affected the 30%DM-70%GGBS specimens regardless of batch volume, causing excessive cracking on the top surface that was the only surface not covered by the specimen mold. An indicative 50 × 50 × 50 mm cubic specimen is shown in Figure 3.

Workability issues were even more profound in larger specimen preparation, especially in the case of full-scale pavement tile development. Poor workability in combination with quick setting led to rushing during placement and casting, which consequently led to specimen imperfections; for example, in some cases the mixture was not adequately placed to completely cover all cube corners. Poor workability also led to difficulties during mechanical property testing, discussed in Section 3.2. The use of silicon powder and/or calcium carbonate auxiliary materials to improve workability, as suggested by previous research studies [36], could be the objective of a subsequent study to specifically address the workability and setting time related challenges faced during this study; this is especially the case when using the geopolymer mixture with the highest GGBS content to cast full-scale pavement tile specimens. Other considerations that are worthy of investigation for optimizing fresh mixture properties would be to further reduce the GGBS content, and/or use alkali activators at lower molarities to activate the raw materials. Further study is required to improve workability and increase the workable time window, thus enabling ease of industrial production that was beyond the scope of this paper.

### 3.2. Hardened Geopolymer Mechanical Properties

#### 3.2.1. Compressive Strength

**Figure 4:**
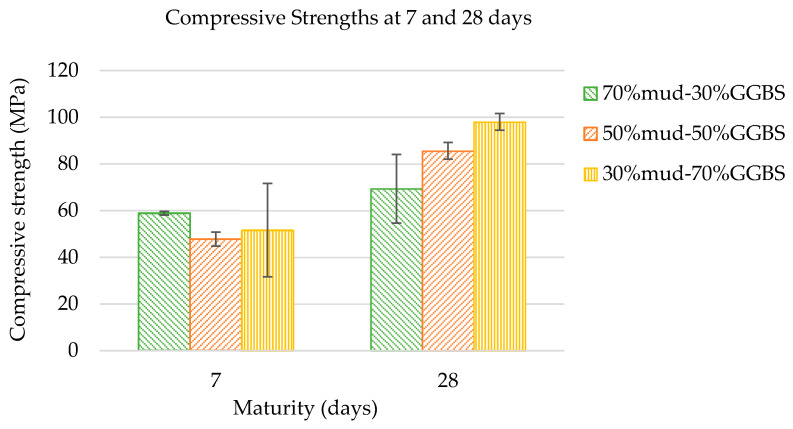
Compressive Strength vs. Maturity for the 3 DM/GGBS mixtures developed.

The compressive strengths reached by the three mixtures developed at 7- and 28-days maturity are presented in Figure 4. The results highlight the ability of the waste DM to reach high compressive strengths when activated in combination with GGBS. As expected, the higher the GGBS content, the higher the compressive strength reached. Most importantly, the fact that a mixture with 70%DM was able to reach an average of 69.38 MPa at 28 days is considered very promising. This clearly indicates that the mixture with the highest DM content (70%DM-30%GGBS) has the potential to be used in construction as a high strength cementless binder, and/or in the development of sustainable precast products, to be used in construction.

First and foremost, utilizing the 70%DM-30%GGBS mixture in construction applications will lead to a significant reduction in the waste DM that is accumulating over the island, and to successful valorization of the DM after a course of 10 years of continuous attempts to reuse it in a cost-effective way.

In cases where higher compressive strengths are required, one of the mixtures that incorporate GGBS at higher quantities could be preferred. However, as revealed by this study, increasing the GGBS content can be detrimental on mix workability. Therefore, further mix optimization is required, including investigation of mixtures with lower GGBS and higher DM content and using a lower molarity activator, especially for large-scale production; that could be the subject of a subsequent study.

#### 3.2.2. Modulus of Elasticity

**Figure 5:**
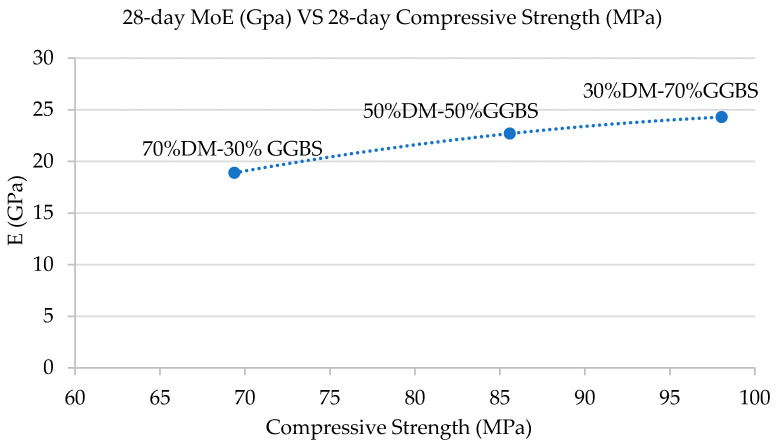
Modulus of Elasticity, E (GPa) vs. Compressive Strength (MPa) at 28-days maturity.

The static modulus of elasticity (E) of the three geopolymer mixtures developed was determined experimentally on cylindrical specimens, loaded as prescribed by the standard procedure that pertains to obtaining the E of concrete at 28-days maturity. The results are presented in Figure 5.

In accordance with the 28-day compressive strengths trend (Figure 4), and as expected, the material E values increased with increasing GGBS content. Considering each mixture compressive strength average at 28 days, the E values obtained are slightly lower than the expected E values of concrete mixtures with similar compressive strengths. Specifically, the 70%DM-30%GGBS mix had a 69.38 MPa average 28-day compressive strength and a modulus of Elasticity at 18.9 GPa. The 50%DM-50%GGBS mix had an 85.58 MPa average 28-day compressive strength and a modulus of Elasticity at 22.7 GPa, and the 30%DM-70%GGBS mix had a 98.05 MPa average 28-day compressive strength and a modulus of Elasticity at 24.3 GPa.

**Figure 6:**
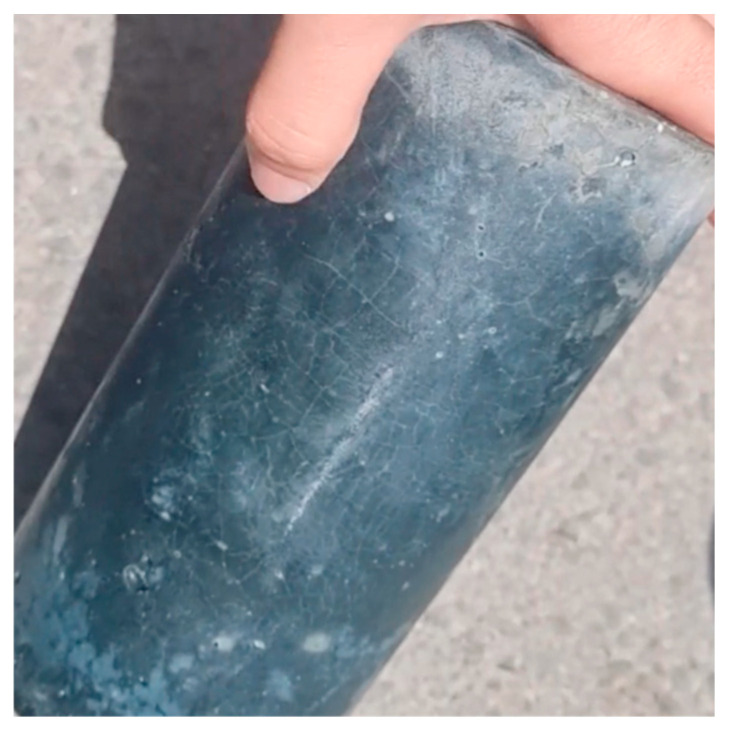
Cracking on Geopolymer Cylindrical Specimen prior to Experimental Testing.

The values are comparable to conventional concrete moduli, considering that the absence of coarse aggregates accounts for lower geopolymer E values. In addition, the cracking observed on the cylinder surfaces before testing, as shown in Figure 6, could be another reason for obtaining lower E values than expected for such high compressive strengths.

#### 3.2.3. Splitting Tensile Strength

The splitting tensile strength of the three geopolymer mixtures developed was experimentally determined at 28-days maturity, following the standard procedure for concrete specimens [29]. The average values obtained per mixture are presented in Table 4. Unlike the compressive strength and modulus of elasticity results, there is no significant disparity between the splitting tensile strength values of the three geopolymer mixtures. The average values obtained by experimentally loading cylindrical specimens from the three mixtures developed in this study to split are within 0.1 MPa. This indicates that the tensile strength will not be affected by increasing the DM content; therefore the mixture that includes greater quantities of DM would be preferred, serving the purpose of sustainability and valorization of the waste DM that has been a prominent source of concern to the local quarry industry and the concrete technology experts on the island for more than 10 years.

#### 3.2.4. Flexural Strength of Prismatic Specimens

**Figure 7:**
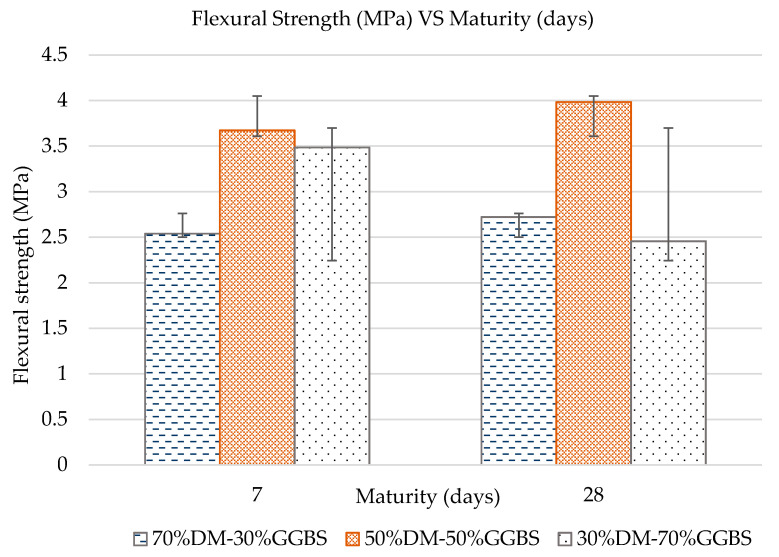
Flexural Strength vs. Maturity per Mixture.

Small prisms were subjected to flexural loading to determine the flexural strength of the three geopolymer mixtures developed, at both 7- and 28-days maturity. The results are presented in Figure 7. The results indicate the superiority of the 50%DM-50%GGBS mixture in terms of flexural strength. The high variability within the obtained results is attributed to pre-existing cracking and specimen imperfections, mostly present on specimens prepared with the 30%DM-70%GGBS mixture, due to limited workability and a short timeframe available for casting and finishing specimens due to quicker mixture setting. In general, the flexural strengths obtained through loading prismatic specimens under flexural bending are not analogous to the compressive strength or the splitting tensile strength values obtained. It is therefore evident that pre-existing cracking is more detrimental to the flexural bending strength, as expected due to the nature of load application during this experimental procedure. As discussed also in Section 3.2.1, a subsequent study could focus on further optimizing the mixture to control the reaction mechanism, which in combination with optimized curing conditions could eliminate pre-existing cracking.

#### 3.2.5. Flexural Strength of Paving Flags

**Figure 8:**
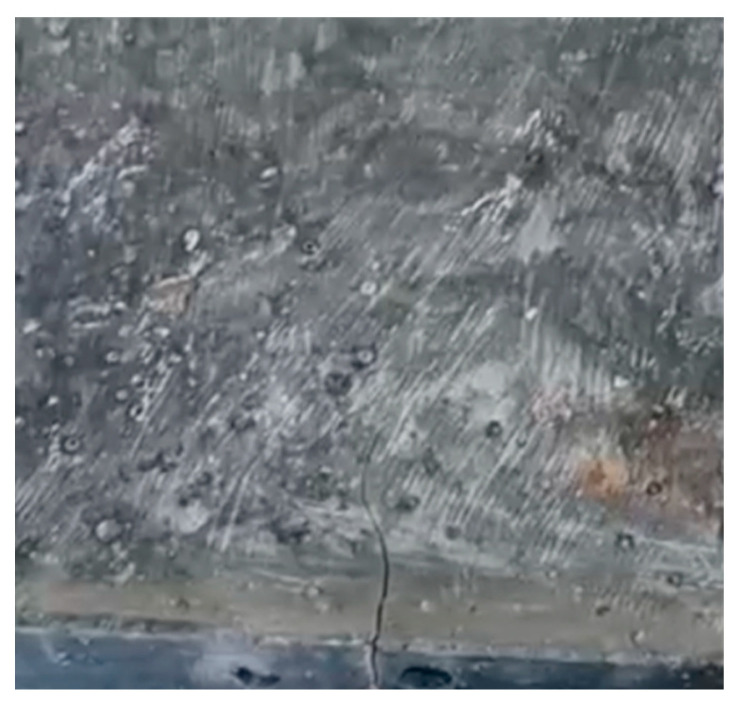
30%DM-70%GGBS Pavement tile specimen image.

**Figure 9:**
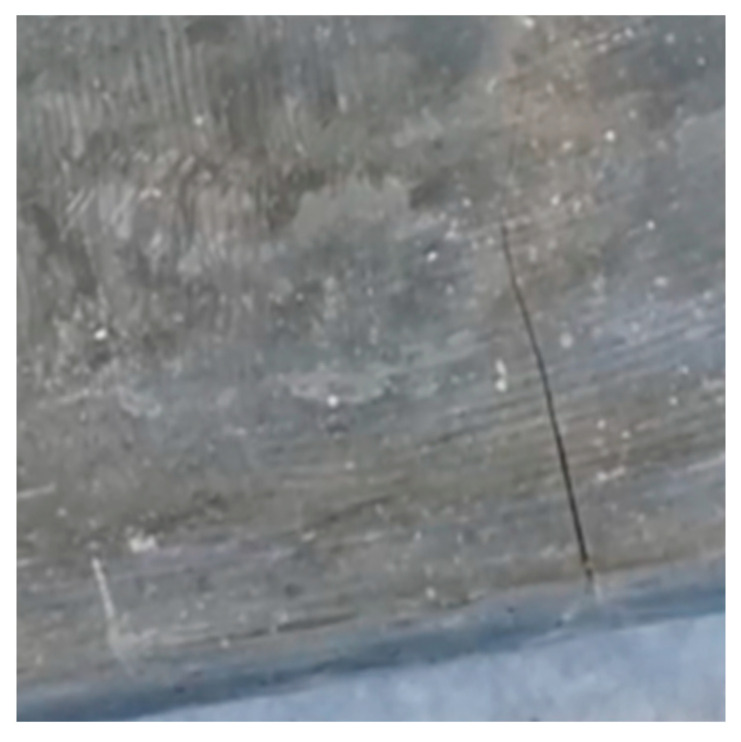
70%DM-30%GGBS Pavement tile specimen image.

Prototype geopolymer pavement tiles were manufactured using the three high performance cementless binder mixtures developed in this study. After 10 years of trials to valorize the local waste DM, this was the first time a precast product was developed using the DM in its original sludge form. The prototype pavement tiles were experimentally assessed under flexural bending at 28-days maturity, following standard procedures for conventional concrete paving flags [32]. The average flexural strength values obtained per mixture are presented in Table 5. The flexural strength values obtained through experimentally loading the prototype specimens under flexural bending are lower than expected, considering other mechanical properties of the developed mixtures. The low flexural strengths are attributed to the preexisting cracking on the pavement tile surfaces, as indicated by specimen images shown in Figure 8 and Figure 9.

Excessive cracking was observed on the tile surfaces before any experimental loading was applied on the specimens. Numerous hairline cracks were visible on the top surface of each specimen, while at least one thicker crack had formed perpendicular to the casting form edge (at least on one side) on all specimens. The multiple hairline cracks are identified as autogenous shrinkage cracking and the wider cracks that formed perpendicular to mold edges are possibly caused by drying shrinkage, since they were always perpendicular to the edges and their width varied between 0.30 and 0.72 mm.

The average flexural strength values obtained by experimentally loading three pavement tile specimens per geopolymer mixture developed, following the standard procedure for concrete pavement tiles are shown in Table 5.

The density of each paving flag tested in this study was determined by accurately weighing each specimen to the nearest gram and precisely measuring the dimensions of each specimen to the nearest tenth of a millimeter. The average density per mixture is presented in Table 6. As expected, the density increases with increasing GGBS content. The pavement tiles developed in this study using the high performance cementless mixtures offer a sustainable, much lighter-weight alternative to conventional concrete pavement tiles, while also being superior in terms of compressive strength.

#### 3.2.6. Sorptivity

Cubic specimens were used to determine the capillary absorption of the three hardened geopolymer mixtures developed in this study. The experimental procedure for measuring the rate of liquid absorption (sorptivity) by hydraulic cement concrete [25] was used.

The results from the capillary absorption test can give a good picture of how susceptible the hardened geopolymer can be to liquid ingression, which will consequently be detrimental to its long-term durability [37].

**Figure 10:**
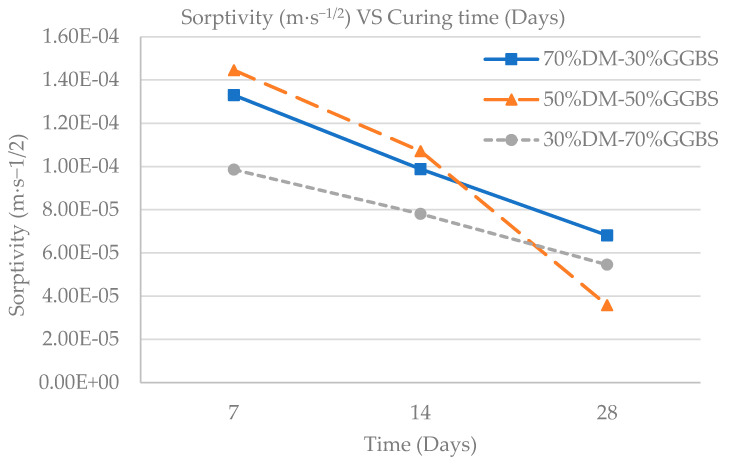
Sorptivity vs. Maturity per Geopolymer Mixture.

The results indicate that increasing material maturity reduces capillary absorption, since the experimental sorptivity values in all three cases decreased between 7 and 28 days, as shown in Figure 10. The values obtained are lower than sorptivity values of conventional concrete specimens [37].

**Figure 11:**
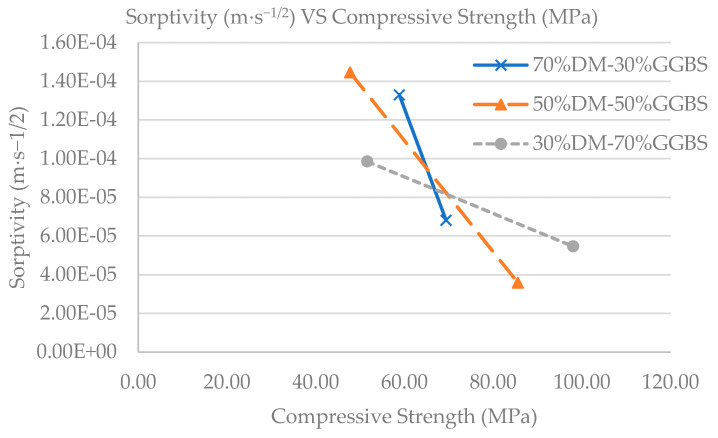
Sorptivity vs. Compressive Strength per Geopolymer Mixture.

In addition, a similar trend in the rate of sorptivity reduction is observed for the 70%DM-30%GGBS and 30%DM-70%GGBS mixtures, indicating similar pore network development. A greater rate of reduction is indicated after 14 days maturity, but only for the 50%DM-50%GGBS mixture. An analogous increased compressive strength was observed in cubic specimens of the same mixture, and both can be explained by reduced pore formation, or improved microstructure. Further investigation of the microstructure of DM/GGBS combination mixtures in a subsequent study is required for confirmation. Figure 11 shows the experimental sorptivity values with respect to the compressive strengths obtained by each developed mixture, at both 7- and 28-days maturity. It should be noted that the S/L ratio was 3.75 for the 30%DM-70%GGBS specimens, while the S/L ratio was 5 for all other specimens (70%DM-30%GGBS and 50%DM-50%GGBS), therefore the microstructure of the mix is affected not only by the varying DM and GGBS content but also by the different S/L ratio of the 30%DM-70%GGBS specimens, which is reflected in both the Sorptivity and compressive strength results.

At 28 days, the lowest sorptivity values were obtained by the 50%DM-50%GGBS mixture while also a greater rate of reduction in sorptivity was achieved by the same mixture between 7- and 28-days maturity. This implies that the addition of GGBS beyond a certain ratio does not improve geopolymer microstructure. Given the fact that GGBS must be imported to Cyprus, the 70%DM-30%GGBS and 50%DM-50%GGBS mixtures attain greater cost-to-performance value. The two mixtures have demonstrated adequate mechanical property performance even though cementless. Most importantly, they are capable of absorbing problematic waste material [20] at a higher content percentage, especially the 70%DM-30%GGBS that utilizes the highest quantity of DM. The specific mixture will be adopted for production, following further technical and economical evaluation (technoeconomic and life cycle analysis).

## 4. Discussion and Conclusions

Successful geopolymerization of local quarry waste DM was accomplished, following a ten-year course of attempts to valorize a waste material that is continuously produced in large quantities at diabase quarries in Cyprus. Adequate DM activation was achieved in combination with GGBS, using 8M NaOH and Na_3_SiO_2_ at a S/L ratio of five and curing under ambient conditions. The results highlight the potential of waste DM in the development of high performance cementless precast products to be used in construction. Even though difficulties were encountered during casting, especially when larger volume mixtures were produced and mostly when the mixture with the highest GGBS content was utilized, adequate mechanical properties were achieved by all three mixtures developed.

Specifically, the 70%DM-30%GGBS mixture reached an average compressive strength of 69.38 MPa, the 50%DM-50%GGBS mixture reached an average of 85.58 MPa, and the 30%DM-70%GGBS mixture reached an average of 98.05 MPa at 28 days. The elastic moduli of the geopolymer mixtures are lower than expected based on compressive strength values. As the GGBS content increased, both the compressive strength and Modulus of Elasticity increased. The three mixtures had similar splitting tensile strength and flexural bending performance. Due to excessive drying shrinkage cracking present on the surfaces of pavement tile specimens cast in this study, the flexural strengths of paving flags obtained are not considered indicative of the developed mixtures. Considering capillary absorption, the three DM/GGBS combination geopolymers outperform conventional concrete in terms of susceptibility to liquid ingression.

To enable efficient large-scale production of sustainable and cost-effective construction products, further mixture optimization and most importantly optimization of curing procedure is required to reduce shrinkage. Utilizing the 70%DM-30%GGBS mixture for the development of industrial products is suggested. Not only did the 70%DM-30%GGBS mix experience the least porosity and least shrinkage cracking out of the three mixtures developed, but most importantly, it is the most sustainable and widely effective of the three since it is comprised of DM to a larger extent. Further research on the developed geopolymer mixture is suggested, as follows:

- Investigating the reaction mechanism and microstructure evolution of alkali activated DM/GGBS combination mixtures would lead to optimizing the fresh properties of the developed geopolymers, thus eliminating specimen surface imperfections and pre-existing cracking, and therefore leading to enhanced hardened geopolymer mechanical properties;
- Optimizing the curing conditions to specifically suit DM/GGBS combination geopolymer mixtures is required, to tackle shrinkage cracking issues caused due to premature drying.

## Figures and Tables

**Table 1:** Diabase Mud Oxide Composition obtained from ED-XRF analysis [19].

	Na_2_O	MgO	Al_2_O_3_	SiO_2_	CaO	ZnO	FeO
%	2.53	8.87	11.14	40.91	5.36	1.71	13.65

**Table 2:** GGBS Technical Data [23].

Property	Value (Unit)
Blaine Specific Surface Area	4450 ± 250 cm^2^/g
Indicative median diameter (d50)	11 μm
Sieve undersize (32 μm)	≥95%
True Density	2.90 ± 0.03 g/cm^3^
Bulk Density	0.8 ± 0.1 g/cm^3^
Color Index [CIE l*ab] with CR410	L* = 89.5 ± 2
Loss on ignition (950 °C)	<1.5%
Water content (100 °C)	<0.5%

**Table 3:** Constituent Content per 1 m^3^ Mixture (3 Mix Designs).

Constituent	70%DM-30%GGBS	50%DM-50%GGBS	30%DM-70%GGBS
DM	1167.04 kg	856.02 kg	472.40 kg
GGBS	500.16 kg	856.02 kg	1102.26 kg
Na_3_SiO_2_	166.72 L	171.20 L	209.96 L
8M NaOH	166.72 L	171.20 L	209.96 L

**Table 4:** Average Splitting Tensile strength per Mixture.

	70%DM-30%GGBS	50%DM-50%GGBS	30%DM-70%GGBS
Average Splitting Tensile Strength (MPa)	4.09	4.11	4.01

**Table 5:** Average Flexural strength of Paving flags per Mixture.

	70%DM-30%GGBS	50%DM-50%GGBS	30%DM-70%GGBS
Average Flexural Strength (MPa)	1.91	2.23	2.41

**Table 6:** Average density of Paving Flag Specimens per Mixture.

	70%DM-30%GGBS	50%DM-50%GGBS	30%DM-70%GGBS
Average density (kg/m^3^)	1904.07	2005.85	2049.23

## Data Availability

Not applicable.
